# French National Protocol for Diagnosis and Care of Calpainopathy (LGMD R1/LGMD D4): consensus guidelines for clinical practice

**DOI:** 10.1186/s13023-026-04279-5

**Published:** 2026-02-27

**Authors:** Gianmarco Severa, Sarah Souvannanorath, Iman Tahiri, Christophe Alimi, Abderhmane Slioui, Luisa Villa, Emmanuelle Salort-Campana, France Leturcq, Nathalie Streichenberger, Martin Krahn, Guilhem Solé, Léonard Feasson, Aleksandra Nadaj-Pakleza, Celine Tard, Tanya Stojkovic, Sabrina Sacconi, Edoardo Malfatti

**Affiliations:** 1 Reference Center for Neuromuscular Disorders, APHP Henri Mondor University Hospital, Créteil, France; 2 Department of Pathology, APHP Henri Mondor University Hospital, Créteil, France; 3 University Paris Est Créteil, Inserm, U955, IMRB, Créteil, F-94010 France; 4 Filière nationale FILNEMUS, Paris, France; 5 Faculté de Santé, Institut Universitaire de Kinésithérapie, Université Paris Est, Ile-de-France, Fontainebleau, France; 6 Peripheral Nervous System and Muscle Department, Université Côte d’Azur, CHU Nice, Nice, France; 7 Neuromuscular and ALS Reference Center PACARARE, La Timone Hospital University, APHM, Marseille, France; 8 Service de Médecine Génomique des maladies de système et d’organe, AP-HP. Centre-Université de Paris, Fédération de Génétique et de Médecine Génomique, Hôpital Cochin, Paris, France; 9 Physiopathologie et Génétique du Neurone et du Muscle, Institut NeuroMyoGène, UMR CNRS 5261 - INSERM U1315, Université Claude Bernard Lyon 1, Lyon, France; 10 Department of Neuropathology, Groupe Hospitalier Est, Hospices Civils de Lyon, Bron, France; 11 INSERM, Marseille Medical Genetics, U1251, Aix-Marseille Université, Marseille, 13385 France; 12 Medical Genetics Department, Timone Hospital, APHM, Marseille, 13385 France; 13 Department of Neurology, Nerve-Muscle Unit, AOC Reference for Neuromuscular Disorders, University Hospital (CHU) of Bordeaux, Pellegrin Hospital, Place Amélie Raba Léon, Bordeaux, 33000 France; 14 Unité de Myologie-Service de Physiologie Clinique et de l’Exercice, Laboratoire Interuniversitaire de Biologie de la Motricité, Centre Hospitalier Universitaire Saint-Etienne, Université Jean Monnet Saint-Etienne, Saint-Etienne, France; 15 Service de Neurologie, Centre de Référence des Maladies Neuromusculaires NEIdF, ERN EURO-NMD, Hôpitaux Universitaire de Strasbourg, Strasbourg, France; 16 Neurology and Movement Disorders Department, Reference Centre for Neuromuscular Diseases, U1172, University Hospital Centre (CHU) de Lille, Nord Est Ile de France, Lille, France; 17 Centre de Référence des Maladies Neuromusculaires Nord/Est/Ile-de-France, Service de Neuromyologie, Institut de Myologie, Hôpital Pitié-Salpêtrière, APHP, Paris, France; 18 Centre de Référence de Pathologie Neuromusculaire Nord-Est-Ile-de-France, Université Paris Est, U955 INSERM, IMRB, APHP, Henri Mondor Hospital, Créteil, France

**Keywords:** Calpainopathy, CAPN3, guidelines, management

## Abstract

**Background:**

Calpainopathies, including limb-girdle muscular dystrophy recessive type 1 (LGMD R1) and the rare dominant type 4 (LGMD D4), are genetic neuromuscular disorders caused by pathogenic variants in the *CAPN3* gene, which encodes calpain-3, a muscle-specific cysteine protease. This protein plays a crucial role in muscle remodelling, calcium homeostasis, and myogenesis regulation.

**Main body:**

LGMD R1, the most common form of LGMD, is characterized by progressive, symmetrical muscle weakness primarily affecting the shoulder and pelvic girdles, with an onset ranging from childhood to adulthood. It affects about 1 in 100,000 individuals. The clinical spectrum is wide, with three main phenotypes: pelvifemoral myopathy, scapulohumeral myopathy, and isolated hyperCKemia. LGMD D4 is characterized generally by a milder phenotype with variable but frequent axial muscle involvement. Diagnostic algorithm include serum CK levels dosage, muscle imaging, and sometimes a muscle biopsy, with definitive diagnosis confirmed by *CAPN3* gene testing. Calpainopathies have no cure and care is focused on physiotherapy, management of muscle contractures, moderate physical activity, and orthosis.

**Conclusion:**

The newly established French National Diagnosis and Care Protocol (NDCP) aim to standardize the diagnosis and care of calpainopathies. The protocol emphasizes early diagnosis, personalized patient care, genetic assessment and prevention of disease progression. This initiative aims to reduce diagnostic delays and improve patient outcomes through a harmonized multidisciplinary approach, informed by the latest clinical and research expertise.

## Background

Calpainopathies are rare genetic muscular dystrophies caused by biallelic (LGMD R1) or, more rarely, monoallelic (LGMD D4) pathogenic variants in *CAPN3* gene, located on the chromosome 15, and leading to the absence or dysfunctional muscle-specific calpain-3 protease in myocytes [1, 2]. They cause progressive, symmetrical muscle weakness with limb girdle and axial distribution [3]. The age at onset ranges from 2 to 40 years [3], and after a mean disease duration of 15 years, most patients with biallelic variants, become wheelchair-bound. Calpainopathies show a wide phenotypic variability, ranging from mild to severe forms, even within the same family or among families carrying the same pathogenic variants [4, 5]. There is no cardiac involvement, while a restrictive respiratory syndrome may occur in the late stages of the disease [6].

## Molecular Basis

In LGMD R1, pathogenic variants can be functionally classified into two major categories: null and non-null variants. Null variants (loss of function), including frameshift and nonsense variants, large deletions, and severe splicing, usually lead to a complete absence of functional calpain-3. Non-null variants, predominantly missense, permit synthesis of a full-length protein but impair its stability, autocatalytic activity, or interactions resulting in a dysfunctional protease [7, 8]. The combination of these variants result in three possible genotypes for LGMD R1: 1) non-null / non-null, biallelic missense (or other non-null) variants resulting in production of full-length protein but with a functional alteration; 2) null / non-null, combining one allele with complete loss of function and one with residual impaired protein function; 3) null / null, biallelic loss-of-function variants, leading to the absence of detectable functional calpain-3. The severity of the clinical phenotype correlates with the underlying genotype. Individuals with a *null/null* genotype, resulting in the absence of protein, typically exhibit an early onset and a more severe phenotype compared with patients harbouring at least one non-null variant [2, 9]. Recent evidence highlights the importance of analyzing the non-coding 3’ untranslated region (3’ UTR) of *CAPN3* in patients with only a single pathogenic variant identified by standard sequencing approaches, as recurrent 3’ UTR variants can act as a second hit, significantly reducing CAPN3 expression and contributing to disease manifestation [10]. In LGMD D4 eleven pathogenic missense variants have been described from 2016 [11–18].

### Epidemiology

LGMD R1 represents the most frequent form of Limb Girdle Muscular Dystrophy in southern Europe, accounting for about 30% of cases of LGMD in France [19]. The recently described autosomal dominant form, Limb Girdle Muscular Dystrophy Dominant type 4 (LGMD D4), is extremely rare [11, 12, 14–18, 20]. In France, the estimated prevalence of LGMD R1 is between 10 and 70 cases per million population [21, 22]. Higher prevalence rates have been reported in genetically isolated communities: 48 per million in La Réunion island and 69 per million in the Basque Country region [22]. At the time of writing this NDCP, the French national calpainopathy registry, coordinated by Prof. Edoardo Malfatti and funded by AFM-Téléthon, is under development and about to start patient enrolment. This initiative will provide more accurate estimates of the incidence and prevalence of this muscular dystrophy in France.

### Pathophysiology

**Fig. 1:**
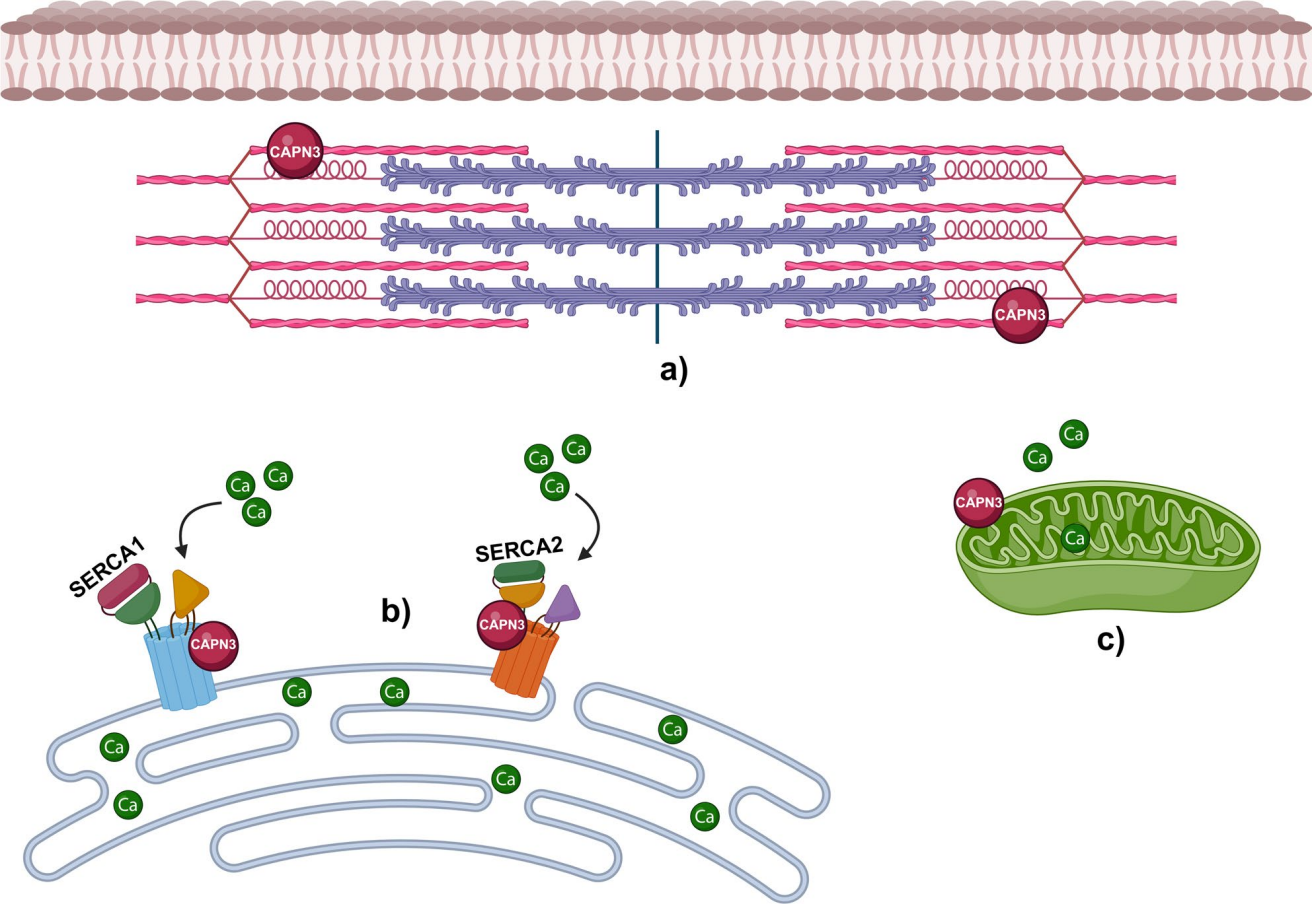
Calpain-3 functions in skeletal muscle. Calpain-3 functions in skeletal muscle. **a**) Sarcomere remodelling and functional interaction with titin. **b**) Sarcoplasmic reticulum: stabilization of calcium-handling proteins such as SERCA1 and SERCA2, with an indirect role in calcium homeostasis. **c**) Effects on mitochondrial function. Figure 1 is realized using biorender.com. license provided by EM

Calpain-3, a calcium-dependent cysteine protease, plays multiple roles in muscle cells not all fully elucidated. Among them: (i) calpain-3 is involved in fiber remodelling. Its function is to degrade damaged or unnecessary proteins, facilitating tissue regeneration [23]; (ii) it contributes to calcium ion (Ca²⁺) homeostasis interacting with calcium-handling proteins in sarcoplasmic reticulum [24, 25]; (iii) calpain-3 is localized in the cytoplasm of muscle cells and it plays a key role in the regulation of myogenesis, contributing to sarcomere size and restoration [26, 27]. In experimental animal models, calpain-3 deficiency has been associated with fiber size variability, mitochondrial dysfunction, impaired energy metabolism, and increased susceptibility to oxidative stress [28]. Recent studies in human muscle have demonstrated that calpain 3 deficiency results in integrin β1D mislocalization and costamere disorganization, with consequent alterations in sarcolemma–ECM coupling, nuclear structure, centrosome localization, and cytoskeletal dynamics [29]. The main calpain-3 functions in skeletal muscle are depicted in Fig. 1.

## Methods and objectives

The objective of this NDCP is to provide healthcare professionals with clear guidelines on the optimal diagnostic and management, as well as the recommended care pathway for patients. The goal is to optimize and harmonize both the diagnostic algorithm and management of the disease, reducing the diagnostic delay. It serves as a practical tool for physicians, particularly when establishing the global care programme in collaboration with patients, their caregivers, and healthcare staff. This protocol aims to: (i) provide the necessary key-points to suspect and confirm the diagnosis of calpainopathy; (ii) define recommendations to slow disease progression and worsening phases; (iii) describe clear guidelines of patient monitoring and follow-up.

This NDCP was developed in accordance with the “Method for Developing a National Diagnosis and Care Protocol for Rare Diseases,” as published by the French Health Authority in 2012 [30] and under the guidance and coordination of the FILNEMUS network. The content was derived from a critical analysis of international literature and the “Gene Review” dedicated to calpainopathy, with relevant bibliographies presented thematically. A multidisciplinary working group wrote and validated the NDCP, and the proposals were reviewed by an additional expert group. The final document was discussed and approved by the multidisciplinary team during a series of conference calls.

### Disease presentation, diagnosis and management

The age of onset is variable usually ranging from 2 to 40 years and it includes: early onset (below the age of 12), classic onset (between 12 and 30 years), or late onset (above the age of 30) [31].

There is no neonatal hypotonia or delay in motor milestones [9]. Patients present with symmetrical and progressive limb girdle muscle weakness followed by axial involvement. More rarely, autosomal dominant cases with very late onset (after 60 years) have been reported with predominant axial involvement (camptocormia) [32].

LGMD R1 can present with three main clinical phenotypes according to the distribution of muscle weakness at onset: (1) Pelvifemoral phenotype: muscle weakness appears first in the pelvic girdle and thigh with major involvement of the posterior compartment (hamstring) and later, at shoulder girdle with *scapula alata*. The onset is variable from early cases to late onset. Individuals with early onset and rapid disease progression typically have a pelvifemoral phenotype [3, 31]. (2) Scapulohumeral phenotype: muscle weakness initially affects the shoulder girdle, often with prominent *scapula alata*, and later extends to the pelvic girdle. Disease onset is frequently later in life. Disease progression is variable but generally slower compared to the pelvifemoral phenotype [3, 31]. (3) HyperCKemia : hyperCKemia can be considered as a presymptomatic stage of calpainopathy, as it is usually observed in children or young adults with recessive calpainopathy, frequently detected during family screening [33, 34].

LGMD D4 was first described in 2016 and currently there are around 100 described cases of dominant calpainopathies in the literature [11, 12, 14–18, 20] The mean age at clinical onset is 34 years, 16 years later than British and Danish cohorts of LGMD R1 [20]. The clinical phenotype is usually milder compared to LGMD R1, including almost asymptomatic patients, myalgia, early Achilles tendon contractures, camptocormia and back pain (present in > 50% of heterozygotes for the *CAPN3* c.643_663del21 pathogenic variant). A small percentage of patients become wheelchair dependent after the age of 60 [11, 14, 15, 18, 20].

### Early stages of the disease

At early stages LGMD R1 classically shows:

- Proximal muscle weakness variably affecting upper or lower limb according to clinical phenotype. At lower limb the posterior compartment of thigh is severely affected also at early stages [35]. Axial weakness is usually present involving paravertebral muscles especially at lumbar level [8, 36].
- Muscle atrophy in the lower limbs primarily involves the gluteal muscles, hamstrings, adductors and calves. In the upper limbs, atrophy affects the *biceps brachii* and periscapular muscles in scapulohumeral phenotype while *triceps brachii* can eventually be involved in pelvifemoral phenotype. Symmetrical *scapula alata* affecting up to 19% of patients at onset, is invariably present in all patients during disease progression [8, 31].
- On physical examination, patients present with a major *scapula alata*, waddling gait, walking on tiptoes and running difficulties associated to hyperlordotic posture with a prominent abdomen due to paravertebral and abdominal muscle weakness.
- Joint contractures may occur during disease progression, affecting the ankles (86%), hips (69%), knees (63%), or elbows (56%) [8]. Rare cases of early onset contractures have been reported [37].
- The facial, ocular, tongue, and neck muscles are typically spared.

The following symptoms can be variably present: back pain and myalgias may appear early also in minimally symptomatic patients [13, 20]; a pseudo-metabolic presentatio*n* with muscle pain and/or exercise intolerance [38]; rhabdomyolysis (and/ or myoglobinuria) triggered by physical activity, occasionally observed in asymptomatic individuals or those with mild muscle involvement [38]; significant calf muscle atrophy or, more rarely, calf hypertrophy [39]; rigid spine syndrome [40]; foot drop [41]; scoliosis [3, 35].

In the advanced stage of the disease, we can observe: difficulty/inability to climb stairs, get up from a chair, get up from the floor, lift weights, or carry heavy loads. Loss of ambulation occurs after a mean period of 15 years following the onset of the disease [19]. In a French natural history study, it was shown that almost half of the population studied (44%) was wheelchair bound: the loss of ambulation occurring at an average age of 28.5 (± 9.5) years [8].

### Muscular and functional assessment

The initial functional assessment of a patient suffering from calpainopathy is performed by a neurologist expert in neuromuscular diseases, a myologist, or a physician specialized in Physical Medicine and Rehabilitation (PM&R) assisted, if possible, by a physiotherapist and/or an occupational therapist. It must include: (1) The functional assessment of transfers, standing, walking, gripping and manual muscle testing. The assessment of walking distance and the number of floors climbed. (2) The evaluation of daily pain and its functional impact. Patient autonomy and walking aid use should be assessed, and falls must be documented and monitored. (3) It is recommended to use standardized functional scores (Walton score, Brooke and Vignos score, MFM scale, NSAD) [42–45] and timed tests (time to climb 4 steps, descend 4 steps, getting up from a chair, Timed Up and Go test, 6-minute walk test), to assess disease trajectory during follow-up.

### Serum Creatine Kinases

The level of serum Creatine Kinase (CK) is 5 to 80 times higher the normal values, from early childhood, especially during the active phase of the disease, with a mean value of 1000–5000 UI/L [46]. In LGMD D4, patients may exhibit normal CK levels [20]. CK level decreases overtime with disease progression due to the fibro-fatty replacement of muscle [47], and frequently reach normal values in wheelchair-dependent patients. A recent study in a small cohort of LGMD R1 patients reported markedly increased urinary N-terminal titin fragment levels compared to age-matched controls, including wheelchair-dependent individuals, suggesting a potential biomarker applicable even in late-stage disease [48].

### Muscle Imaging

**Fig. 2:**
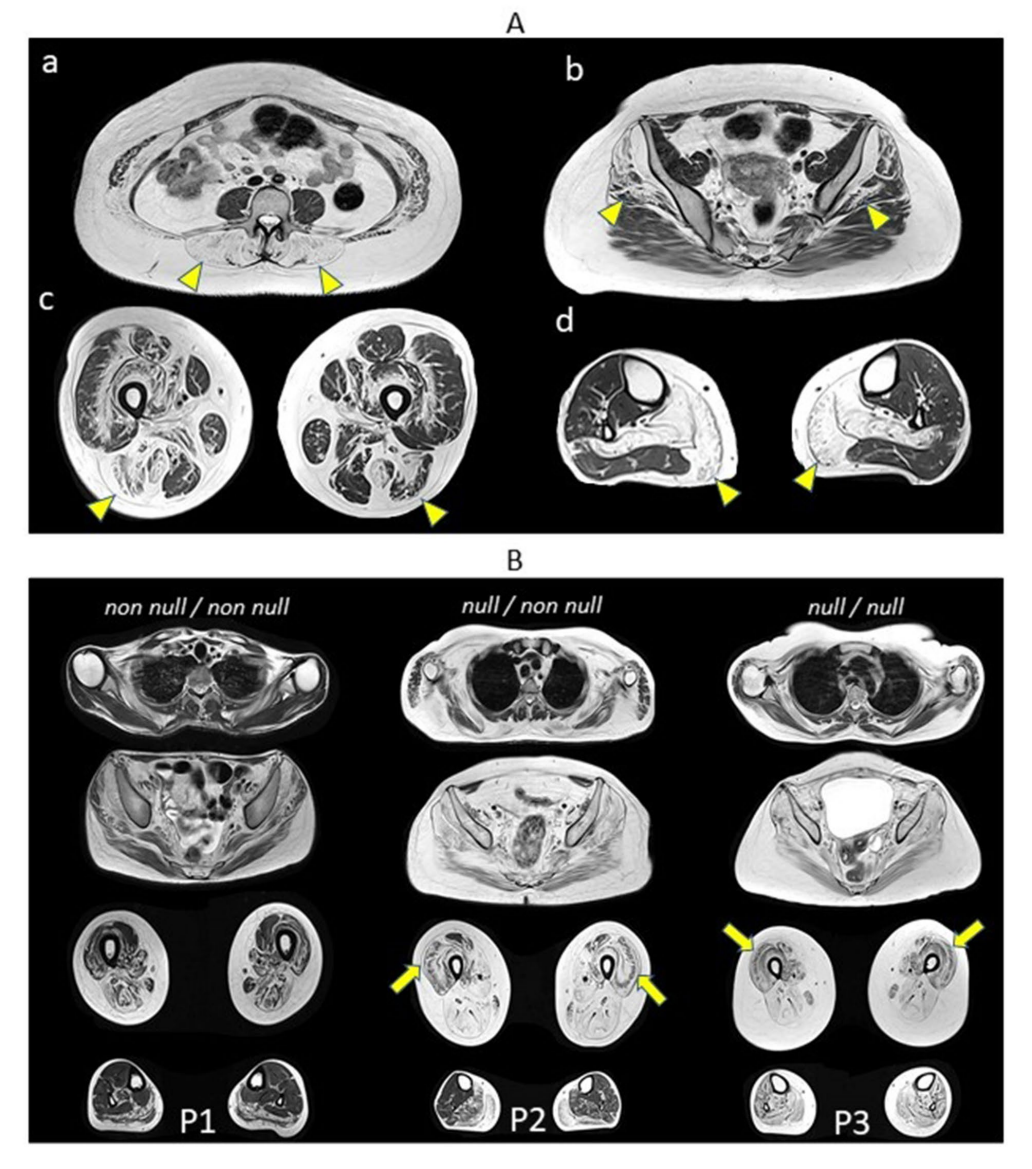
Muscle imaging features of LGMD R1. Axial plane T1 sequence. Panel A (**a, b, c, d**). (**a**) Lumbar level: severe and symmetrical fibro-fatty replacement of paravertebral muscle (yellow arrowheads). (**b**) Pelvis: major and symmetrical fibro-fatty substitution of *gluteus minimus* and *gluteus medius* (yellow arrowheads) with partial sparing of *gluteus maximus*. (**c**) Thigh: bilateral involvement of hamstrings (yellow arrowheads), *vastus intermedius* and partial involvement of *vastus lateralis*. (**d**) Leg: global and symmetrical fibro-fatty replacement of *soleus* and *gastrocnemius medialis* muscles (yellow arrowheads). Panel B (P1, P2, P3). P1) non null/non null genotype and 27 years of age; p2) null/non null genotype and 28 years of age; p3) null/null genotype and 31 years of age. Pseudo-collagen sign at *quadriceps* is indicated by yellow arrow

The main muscle MRI features of LGMD R1 are shown in Fig. 2.

Whole-body muscle MRI or lower limb muscle MRI are recommended in order to characterize and quantify the muscle involvement [35]. During the early stages of the disease, the classical pattern of fibro-fatty replacement in LGMD R1 shows:

- At pelvic level: an early and symmetric involvement of *gluteus minimus* and *gluteus medius* compared to a milder involvement of *gluteus maximus* and a relative sparing *ileopsoas.*
- At thigh level: an early and major involvement of the posterior compartment (*semitendinosus*,* semimembranosus* and long head of *biceps femoris*) and the medial compartment (*adductor magnus*) is observed. In the anterior compartment the *quadriceps* is frequently involved with a partial sparing of *rectus femoris.*
- At leg level: there is a predominant involvement of the *gastrocnemius medialis* and *soleus* while *gastrocnemius lateralis* is less affected. The anterolateral compartment, *tibialis anterior* and the *extensor digitorum longus*, is spared, especially at early stages.
- * At the axial level*: a severe involvement of the *extensor spinae* muscles with relative sparing of the spinal rotators can be found, leading to the hyperlordosis.
- STIR sequences can show hypersignal compatible with the presence of myoedema [49, 50].

In the dominant form (LGMD D4), the paraspinal muscle are frequently and severely involved, especially at lumbar level [20, 32].

The severity of fibro-fatty substitution correlates with the *CAPN3* genotype: in particular, patients with no ‘null’ variant or a single ‘null’ variant exhibit a minor muscle replacement compared to patients with a double null genotype [35]. The severity of muscle involvement according to genotype is depicted in Fig. 2 comparing patients with different genotype, similar age at muscle MRI and similar disease duration. Patients with a longer and more severe course of the disease, can present the pseudo-collagen sign characterized by a relative sparing of the central portion of the muscle [35].

### Muscle Biopsy

**Fig. 3:**
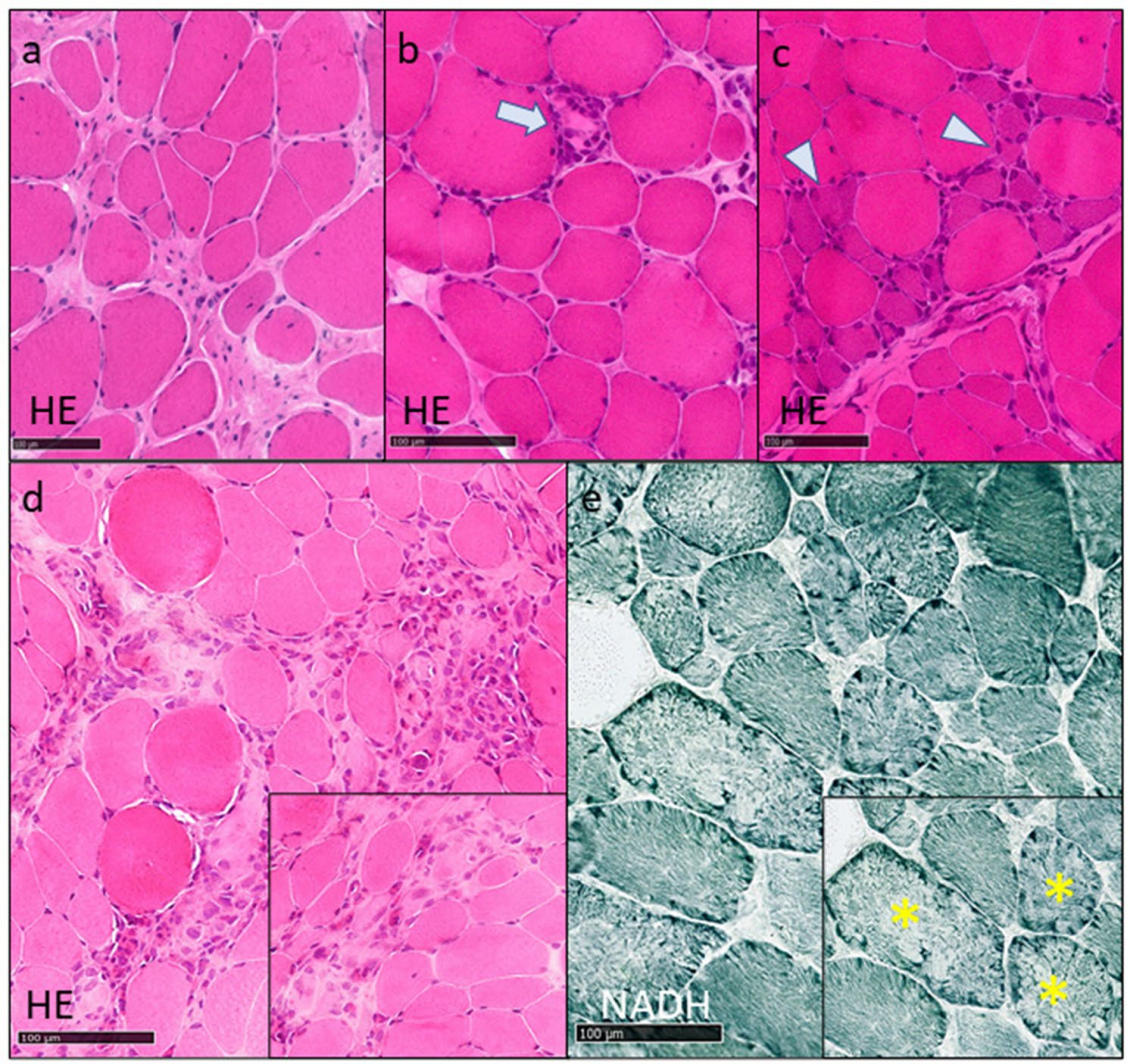
Myopathological lesions in calpainopathy. HE (Hematoxylin and eosin), NADH-TR (Nicotinamide adenine dinucleotide tetrazolium reductase). **a**) fiber size variability, nuclear internalizations and endomysial fibrosis; **b**) fiber size variability and necrosis (blue arrow); **c**) clusters of basophilic regenerating fibers (blue arrowheads); **d**) inflammatory eosinophilic infiltrate; **e**) lobulated fibers (yellow asterisks)

Muscle biopsy is an invasive procedure requiring high expertise, and the techniques are performed in a pathology department affiliated with an expert myopathology center. Clinical examination and muscle imaging guide the selection of the biopsy site; to obtain informative samples, the chosen muscle normally shows an intermediate degree of involvement. Muscles with huge fibro-fatty replacement on imaging should be avoided. The quadriceps and deltoid are typical sites for muscle biopsy, based on the patient’s clinical phenotype. Muscle biopsy findings in a patient with calpainopathy are reported in Fig. 3.

As the diagnosis of calpainopathy is confirmed by molecular genetic studies, a muscle biopsy should be performed: to validate novel variants of uncertain significance (VUS), or in patients showing atypical presentation [51].

The main myopathological lesions are that observed in a myopathic pattern including fibers size variation, nuclear internalization or a more prominent dystrophic pattern with necrotic-regenerating fibers and variable degree of endomysial and perimysial fibrosis.

The oxidative histoenzymatic reaction show frequently an uneven disposal of the mitochondrial and reticular components with clusters of lobulated fibers (Fig. 3).

Inflammatory infiltrates with eosinophils may be present in some cases, and occasionally in the form of eosinophilic myositis [52–54]. In the absence of significant inflammatory infiltrate, markers such as HLA-1 and HLA-2, as well as membrane attack complex (MAC), are usually negative.

Calpain-3 is a relative fragile muscle protease, it is unstable and prone to degradation. No commercial antibody is currently available for its detection in immunohistochemistry.

### Western blot

Western blot analysis of calpain-3 from biopsy-derived frozen muscle tissue is useful, as it allows the quantification of its three calpain-3 isoforms: (i) full-length calpain-3 (94 kDa); (ii) major autolytic fragment (60 kDa); (iii) minor autolytic fragment (30 kDa). Western blot analysis specifically focus on the assessment of the presence of the 94 kDa and 60 kDa isoforms [2]. Interpretation of the western blot analysis can be challenging and is highly dependent on the quality of muscle preservation. Three main conditions can be observed: (1) the absence of both 94 kDa and 60 kDa isoforms suggestive of a LGMD R1. (2) the absence of only one isoform (94 kDa or 60 kDa) or only a mild reduction of both, secondary to a primary defect of dysferlin, titin, or FKRP [55–57]. (3) A normal calpain-3 expression on western blot in a patient with compatible phenotype or muscle imaging can be due to variants on the autocatalytic site and suggest the presence of a normal amount of inactive calpain-3 in about 20–30% of cases [Dr France Leturq personal data] [58].

### Electroneuromyography

ENMG can easily distinguish calpainopathy form insidious neurogenic disorders with proximal muscular weakness such as type 3 spinal muscular atrophy, when clinical phenotype is not clear. In calpainopathy motor and sensory nerve conduction velocities are normal, while the detection highlights a classic myopathic pattern with small polyphasic potentials, however the detection may be normal, especially in asymptomatic or paucisymptomatic patients.

### Differential diagnosis

Among the autosomal recessive forms of LGMDs, calpainopathy should be considered in the differential diagnosis with:

- LGMD R2 (*DYSF*) [59]. Note that there is no *scapula alata* in dysferlinopathies. Whole body muscle MRI shows an early involvement of *subscapularis* and *latissimus dorsi* with less severe involvement of glutei compared to LGMD R1 [60].
- LGMD R9 (*FKRP*) showing proximal upper and lower limb weakness, associated to calves hypertrophy and sometimes tongue hypertrophy. Cardiac involvement in form of dilated cardiomyopathy and a restrictive respiratory syndrome are common [61]. Muscle MRI revealed an early involved of *vastus intermedius*, *biceps femoris* and the *adductor magnus* with relative sparing of the *rectus femoris*, *sartorius* and *gracilis* [62].
- The LGMD R12 *(ANO5)*, presenting as slow progressive LGMD with high CK levels, without major cardiac or respiratory involvement. Muscle weakness and atrophy may be asymmetric. Pseudometabolic forms with episodes of rhabdomyolysis and myalgia with hyperCKemia have been reported [63, 64].
- Some cases of sarcoglycanopathies without cardiac involvement (LGMD R3, LGMD R4, LGMD R5, LGMD R6) [65]. *Adductors*, glutei muscle, and hamstrings present an early and severe involvement in muscle MRI. Quadriceps shows a peculiar proximo-distal gradient of fibro-fatty replacement [66].

### Other myopathies

 Facioscapulohumeral muscular dystrophy (FSHD1) shares some clinical and laboratory features with scapulohumeral phenotype of LGMD R1 [67] such as scapular girdle muscle weakness with *scapula alata*, elevated serum CK levels, and non-specific myopathic changes often observed in muscle biopsy. Facial muscle weakness, an asymmetric involvement, and lower CK level are typical in FSHD.

- Dystrophinopathies comprise a spectrum of muscle diseases caused by pathogenic variants in the *DMD* gene, encoding the dystrophin protein [68]. Arikawa et al. showed how in a cohort of 41 patients with a clinical diagnosis of LGMD, 17% had a dystrophinopathy: five man with a Becker muscular dystrophy and two female patients with heterozygous dystrophinopath*y* [69]. Some cases of Becker muscular dystrophy may be considered in the differential diagnosis; however, the presence of predominant quadriceps atrophy, cardiac and respiratory involvement, calf and tongue hypertrophy, and possible cognitive impairment helps to distinguish the two diseases [70].
- Metabolic myopathy, in particular late onset Pompe diseas*e* (LOPD). Calpainopathy has been reported in people with asthenia, myalgia, exercise intolerance, proximal lower limb muscle weakness, and excessive lactate production after aerobic exercise [40, 71]. LOPD present some myopathological hallmark such as the presence of multiple subsarcolemmal or intermyofibrillar vacuoles filled with PAS positive material (glycogen storage).

The association between rhabdomyolysis and LGMD is less recognized than the association between rhabdomyolysis and metabolic myopathies (i.e., CPT2 deficiency); this often leads to misdiagnosis or diagnostic delay. Some people with calpainopathy have experienced episodes of rhabdomyolysis, mild muscle weakness, and persistent elevation of CK levels even after a myoglobinuric episode (whereas in metabolic myopathies, CK levels between episodes are usually normal) [38].

The main differential diagnosis for calpainopathy are reported in Table 1.

**Table 1:** Calpainopathy differential diagnosis

Myopathy	HyperCKemia	Specific laboratory findings	Distribution of weakness	Symmetrical involvement	Scapular winging	Tendon contractures	Hyperlordosis	Respiratory involvement	Cardiomyopathy	Other involvement
LGMD R1 (C*APN3*)	Yes	NA	Axial, girdles	Yes	Yes	Yes	Yes	Possible, late stage	No	Stiff spine
LGMD D4 (*CAPN3*)	No/variable	NA	Axial, girdles	Yes	Possible	Rare	Yes	No	No	Camptocormia
Immune-mediated necrotizing myopathies (IMNM)	Yes	Anti-HMGCOAR and anti-SRP antibodies (seropositive cases)	Axial, girdles	Yes	No	No	No	Possible	Possible	Subacute evolution
FSHD1	Variable	NA	Facial-Scapular-Humeral-Peroneal	No	Yes	No	Yes	Possible	No	Retinopathy Hearing impairment Epilepsy
Dystrophinopathies (*DMD*)	Yes	NA	Proximal-Diffuse	Variable	Yes	Yes	Yes	Yes	Yes	Cognitive involvement Calf hypertrophy Tongue hypertrophy
Sarcoglycanopathies	Yes	NA	Proximal, pelvic and scapular girdles	Yes	Yes	Yes	Yes	Possible	Yes	Rapid progression compared to other LGMDs
LGMD R9 (*FKRP*)	Yes	NA	Scapular and pelvic girdles	Yes	Yes	Yes	Yes	Yes	Yes	Calf hypertrophy Tongue hypertrophy
LGMD R12 (*ANO5*)	Yes	NA	Proximo-distal	Variable	No	No	No	No	Rare	Pseudo-metabolic form
Late onset Pompe disease (*GAA*)	Yes	Acid maltase dosage	Proximo-distal, scapular and pelvic girdles	Yes	Yes	Yes	Yes	Yes	No	Central vascular involvement

NA: not applicable. FSHD1: Facioscapulohumeral Muscular Dystrophy type 1

### Molecular Genetics

In France the filière neuromusculaire FILNEMUS prioritize a gene panel approach as multigene panel including the *CAPN3* gene and other genes of interest (see Differential Diagnosis ) [72]. Clinicians can choose the appropriate panel based on the patient’s phenotype and paraclinical findings. The genes included in the panel and the diagnostic sensitivity of the test used vary depending on the laboratory and are likely to change over time.

A more complete genomic analysi*s* including exome and genome sequencing can be considered. Compared to direct sequencing, more comprehensive genomic tests were able to improve the detection rate of calpainopathies [73]. Several studies indicate that this test is a cost-effective approach for the diagnosis of calpainopathies [54, 55].

Sanger sequencing may be recommended if a more comprehensive multigene panel and/or genomic testing is not available and the clinical and paraclinical phenotype of the patient is suggestive of calpainopathy. A targeted analysis of certain specific pathogenic variants can be carried out first in individuals from certain populations/origins.

Studies focusing on the phenotype-genotype correlation in LGMD R1, showed how the severity of the clinical phenotype is related to the characteristics of the pathogenic variants. Patients with two null variants, resulting in a premature truncation of protein synthesis, present a more severe phenotype and an early onset compared to patients with at least one non null variant [74–76].

**Fig. 4:**
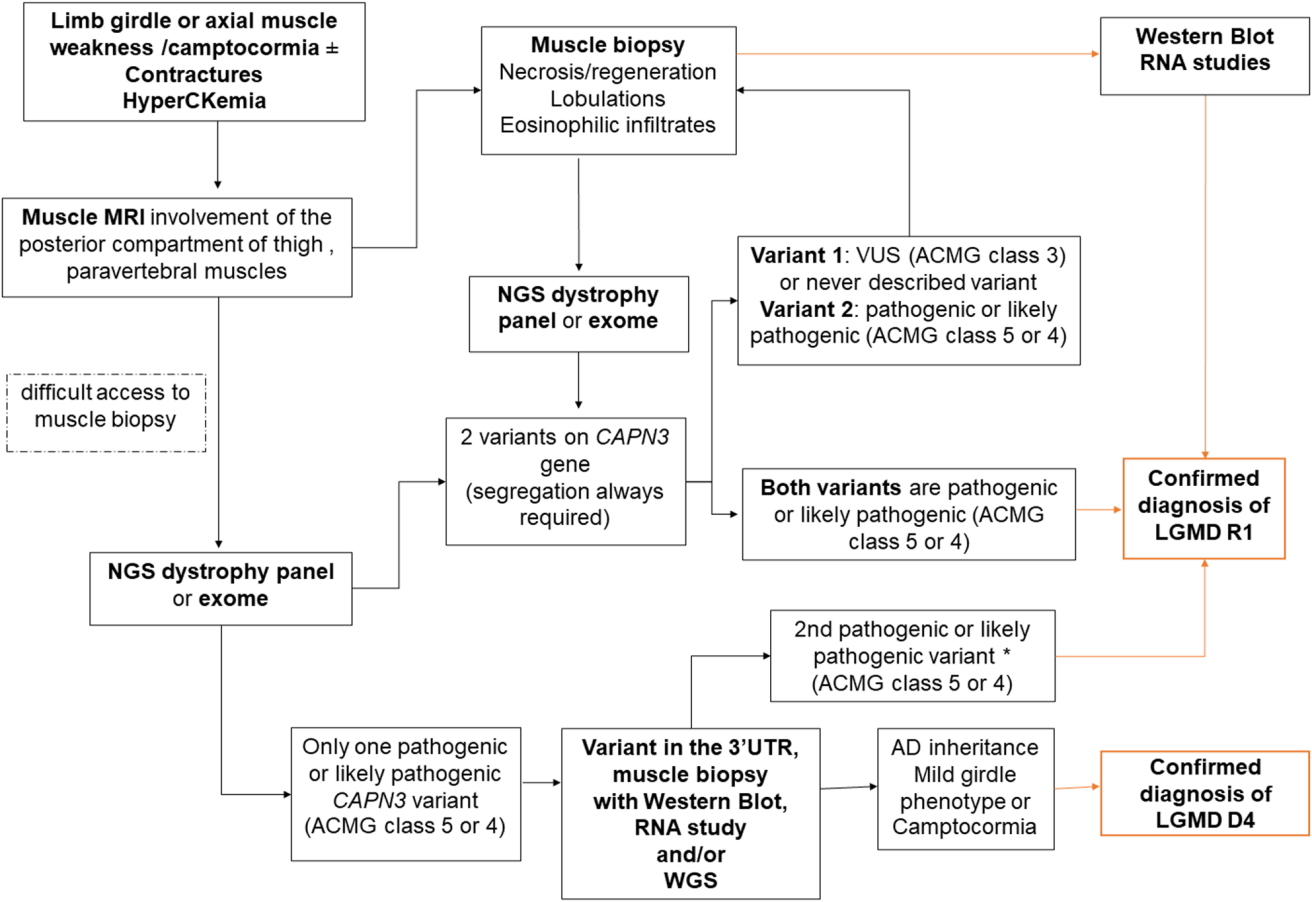
Diagnostic workflow for calpainopathy. Schematic representation of the stepwise diagnostic approach for patients presenting with limb girdle or axial muscle weakness or camptocormia associated with hyperCKemia and/or contractures. The workflow integrates clinical features, muscle MRI findings, genetic testing, muscle biopsy, and functional studies (Western blot and RNA analysis) to distinguish between LGMD R1 and LGMD D4. * the 2nd pathogenic or likely pathogenic variant such as an intronic variant or a VUS (class 3) reclassified as class 5

A complete diagnostic workflow for calpainopathy (LGMD R1/ LGMD D4) is proposed in Fig. 4.

## Search for comorbidities

### Respiratory assessment

The clinical evaluation systematically looks for symptoms and signs related to respiratory failure: sleep disorders (frequent nocturnal awakenings, excessive daytime sleepiness, non-restorative sleep, headaches or morning sickness), dyspnea, orthopnea. Respiratory assessment must be carried out with simple spirometry with measurement of forced vital capacity (FVC) and compared to theoretical values, measurement of maximum inspiratory and expiratory pressures (MIP, MEP). An arterial blood gas analysis is rarely necessary. Polysomnography is recommended for patients with sleep disorders, if not available a night-time oximetry can be performed. Overnight pulse oximetry is recommended if FVC is less than 70 − 60% of predicted values and demonstration of nocturnal hypoventilation should lead to nocturnal non-invasive ventilation (NIV) [77].

### Cardiac assessment

In calpainopathy there is no cardiac involvement, neither as cardiac rhythm abnormalities or conduction disturbances, nor as cardiomyopathy. Assessment of vital signs (heart rate, rhythm, and blood pressure) and a standard electrocardiogram (ECG) is routinely performed. Cardiac ultrasound, a Holter ECG or a stress test may be recommended if clinically indicated.

### Assessment of fracture risk

Patient care must include an assessment of the risk of osteoporotic fracture by bone densitometry. If a risk of fracture is identified, vitamin D levels should be assessed annually, followed by supplementation if necessary and monitored sun exposure.

### Nutritional assessment

Weight gain can worsen functional abilities and increases pain. Insidious weight loss should also be detected, particularly during phases of disease progression, to prevent malnutrition and related complications.

### Progression and variability

LGMD R1 is a slowly progressive disease. A natural history study carried out in France by Richard et al. 2016 [8] in 85 LGMD R1 patients, showed that the deterioration of muscle strength as assessed by the Manual Muscle Testing (MMT) leads to a decrease of 2 to 3% per year compared to baseline, which corresponds to a loss of one half test point in 4 years. The muscles evaluated included mainly proximal lower limb muscles (*psoas*,* quadriceps*, hamstrings, *gluteus medius*) and selected upper limb muscles (*deltoid*,* biceps*,* triceps*), reflecting the typical calpainopathy profile.

The asymptomatic stage may be relatively long in some affected individuals, particularly women. In some people with calpainopathy, the onset or worsening of symptoms may be influenced by environmental factors, such as infectious diseases, strenuous exercise, drug treatments, a traumatic event, or pregnancy [78].

The disease is invariably progressive and loss of ambulation occurs approximately 15 years after the onset of symptoms [5, 9, 78–80]. In general, loss of ambulation occurs earlier in people with childhood onset and double null genotype [81].

More rapid progression of the disease has been observed in men than in women [82]. In a clinical study on the natural history, a higher proportion of women still walked compared to men (72% versus 48% in men) [79]. Men usually show more fiber atrophy, muscle weakness and clinical disability compared to women [81].

Clinical phenotype may present an intrafamilial variability also in siblings with the same pathogenic variant, the age of onset and clinical course can vary considerably [4].This suggests an influence of the genetic background and environmental factors in disease progression.

## Current Management

### Physiotherapy

The lack of disease-modifying treatment increase the importance of preventive, supportive and compensatory approaches [78]. Various studies have demonstrated the long-term benefits of physical exercise in patients with neuromuscular diseases [83]. The physiotherapy protocol must be adapted to each patient, according to capacities and fatigability with a recommended timing of twice weekly for 30 to 45 min each session. Concentric contraction exercises are recommended for controlled muscle strengthening, while eccentric contractions should be avoided in muscles with an MRC rating below 4 due to increased risk of damage. Physiotherapy protocol must include stretching exercises to prevent contractures. This can be associated with mobilizations of the 4 limbs and the trunk. Contractures of the Achilles’ tendon, have to be verified also at early stage of disease [84].

Static and dynamic balance exercises with postural control work, help maintaining patient’s autonomy. This last type of exercise can be performed in a seated position in patients wheelchair bounded with an increased risk of scoliosis. The use of seated positioning devices or orthoses may also be considered.

At the cardiovascular level, gentle, low-impact aerobic exercises can be carried out [84]. The objective will be to fight against deconditioning linked to a sedentary lifestyle. Depending on the patient’s abilities, they may be referred to certified Adapted Physical Activity (APA) instructor for exercise reconditioning through aerobic endurance exercises. Adapted Physical Activity can be provided under supervision to encourage independent exercise. It should be gentle, regular and slowly progressive.

Regarding the management of different types of subacute and/or chronic pain, massage techniques or transcutaneous neurostimulation devices (TENS) may be used for analgesic action.

### Occupational therapy

A global and multidisciplinary assessment (PM&R doctor, occupational therapist, physiotherapist) is essential as soon as functional discomfort appears, especially if there is an impact on daily autonomy (moving, washing, dressing, eating, but also writing, using a computer keyboard, etc.).

The assessment will address several areas, depending on disease severity: daily living aids, travel assistance devices, living space arrangements, evaluations for driving and potential vehicle modifications, as well as adjustments to workplace layouts.

### Surgical management

In general, tendon contractures do not require surgical correction to maintain walking for as long as possible. In specific cases, it is useful to discuss collectively with orthopaedic surgeons to take care of foot deformities or scoliosis.

### Ventilatory support

In advanced disease, chronic restrictive respiratory syndrome may occur and the use of respiratory aids may be indicated to prolong survival.

- Intervention in the form of nocturnal respiratory assistance in cases of respiratory involvement including patients with clinical signs and symptoms of hypercapnia (daytime sleepiness, sleep disturbances, morning headaches) by non-invasive positive pressure ventilation, NIV, or by nasal mask [85, 86].
- People in wheelchairs may also develop weak cough, leading a risk for atelectasis, pneumonia, and respiratory failure.
- High dose oxygen therapy should be avoided without ventilatory support. In calpainopathy, as in other neuromuscular disorders, the primary cause of respiratory impairment is alveolar hypoventilation rather than hypoxemia. Oxygen supplementation without ventilation does not remove CO₂ and may precipitate hypercapnic coma [87, 88].
- Annual vaccination and rapid treatment of lung and respiratory infections should be handled using a mechanical cough-assist device [85].

### Nutrition

Calpainopathies usually do not present dysphagia or secondary nutritional impairment. However, it is important to ensure a balanced diet in order to limit weight gain which can worsen the motor deficit.

## Special situations

### Anaesthesia

No specific recommendations have been published for calpainopathies. As in other muscular diseases, local and regional anaesthesia should be preferred over general anaesthesia to limit the risk of respiratory complications such as aspiration pneumonia, atelectasis or bronchial infection. Respiratory decompensation may occur especially in cases of thoracic or abdominal surgery. In case of general anaesthesia, a prior assessment of FVC is mandatory. Neuromuscular blocking agents with a long half-life such as pancuronium should be avoided because they appear to increase the risk of respiratory complications compared to agents with a shorter duration of action (atracurium or vecuronium). Likewise, rocuronium seems to increase the risk of postoperative respiratory decompensation. There is no strict risk of malignant hyperthermia. On the other hand, suxamethonium chloride should be avoided because of a potential risk of hyperkalaemia and rhabdomyolysis.

### Pregnancy

LGMD R1 does not appear to affect pregnancy and childbirth. Epidural anaesthesia appears to carry less respiratory risk compared to general anaesthesia.

### Vaccination

There are no vaccine contraindications. However, in patients with calpainopathy presenting muscle weakness, it is preferable to vaccinate in a poorly affected muscle or subcutaneously.

### Registries

Patient registries are key elements in the advancement of knowledge and science, especially regarding prevalence, natural history, pathogenesis and treatment options of the disease. They also promote close collaboration between patients, healthcare professionals, academia and industry, creating a strong interaction for research and patient care. Additionally, these registries provide an accurate picture of the true disease burden for targeted cohorts, standards of care, and patient treatment preferences. Several registers have been set up for muscular diseases, two of which focus specifically on calpainopathies: the Registry of the Coalition to Cure Calpainopathies (international) and the National Registry of Calpainopathies of the AFM Téléthon in France [21].

This French national register on calpainopathies is being developed under the leadership of Dr. Isabelle Richard and Prof. Edoardo Malfatti. This project aims to deepen knowledge of the disease, its epidemiology and its natural evolution by collecting data from 23 neuromuscular centres affiliated with the national FILNEMUS network. The resulting analyses and subsequent studies will help define the key endpoints for future clinical trials, including those involving gene therapies.

## Ongoing therapeutic strategies

### AAV-mediated gene therapy

In LGMD R1 mouse model, AAV-mediated *CAPN3* gene transfer has shown safety and efficacy, leading to a significant myopathological improvement. However, recent AAV-based clinical trials in other inherited myopathies have reported severe adverse events, including acute liver failure, highlighting the importance of careful safety assessment and monitoring in systemic AAV gene therapy approaches [89]. An effective gene vector to restore calpain-3 in skeletal muscle have been developed but the experiments showed a major cardiac toxicity [90]. Recently a mechanism able to explain the different regulation on calpain-3 activity in the heart and in skeletal muscles have been identified. Last studies showed how the regulation of calpain-3 activity in skeletal muscle is influenced by a region of titin. This advance makes it possible to design gene therapies that specifically target skeletal muscles, avoiding any toxic effect on the heart [91, 92].

In addition, Nationwide Children’s Hospital recently announced a gene therapy program using the AAVrh74 vector, designed to replace *CAPN3* in skeletal muscle via systemic administration. AAVrh74 has a strong tropism for muscle cells and a relatively low level of pre-existing immunity. This vector has already been used in other gene therapy programs targeting Duchenne muscular dystrophy and five other LGMD subtypes [89, 93].

### Cell Therapy

Cell-based therapies for calpainopathy are a rapidly evolving with promising advances. Recently researchers corrected *CAPN3* gene c.550delA using CRISPR/Cas9 technique in human induced Pluripotent Stem Cells (hiPSC) and in Primary Human Satellite Cells (PHSat). Edited cells demonstrated restored calpain-3 protein expression, maintained myogenic potential, and successfully formed functional myotubes in vitro and in vivo. Cell therapies, whether autologous or allogeneic, offer new perspectives for treating calpainopathy and other forms of muscular dystrophy [94].

### Other pharmacological approaches

Pharmacological approaches aim to slow the progression of the disease. Several drug candidates are being tested. In recent years, a research group conducted a study on human *CAPN3*-mutated myotubes treated with bortezomib (a proteasome inhibitor), showing an increase in SERCA2 in the sarcoplasmic reticulum along with normalization of basal intracellular Ca²⁺ levels. However, no changes in SERCA2 levels were observed after bortezomib treatment in the *CAPN3*- knockout mouse model [95]. A study focused on the effect of calpain-3 deficiency in mitochondrial function, investigated the use of GW501516, a PPAR-delta agonist, to improve mitochondrial biogenesis. They treated with GW501516 a 7 month old *CAPN3*-knockout mouse model showing improved satellite cell activity and reduced muscle fatigability and serum creatine kinase levels [96]. Tideglusib, a GSK-3β inhibitor, has emerged as another potential pharmacological approach for LGMDR1. In vitro studies in patient-derived myotubes have shown that Tideglusib restores the activity of the Wnt and mTOR pathways, increasing β-catenin levels. These effects may contribute to the maintenance of costamere structure and improved muscle fiber function, potentially reducing degeneration [97]. Additional studies are needed to evaluate the safety profile of this approach.

Since 2015, the I-stem laboratory has been working on model cells for limb-girdle muscular dystrophies to identify drug candidates among already known molecules [98, 99].

One proposed pharmacological approach is to increase muscle mass by enhancing positive regulators of muscle growth, or inhibiting negative regulators, such as myostatin. In the *CAPN3* ^−/−^ mouse model of LGMD R1, treated with AAV-carrying a mutant myostatin propeptide with an inactive C-terminal domain showed increased muscle mass and improved muscle strength. On the other hand, a phase I/II trial with MYO-029, a recombinant human antibody that inhibits myostatin activity showed good tolerability but minimal improvement in muscle strength and pathology of patients with LGMD [100].

## Conclusion

Calpainopathy affects approximately 1 in 100 000 patients worldwide. The disease is characterized by a progressive symmetrical muscle weakness with limb girdle and axial distribution. The main clinical phenotypes are described according to the distribution of muscle weakness at onset, as the pelvifemoral and scapuloperoneal forms, both typically associated with *scapula alata* with disease progression. Respiratory involvement, in the form of a restrictive respiratory syndrome, may appear in later stages. Cardiac involvement is absent. Skeletal muscle weakness and motor impairment represent the main sources of functional difficulties for patients.

Currently, there is no specific disease-modifying treatment. Management strategies are primarily centred on targeted physiotherapy protocols. Regular clinical follow-ups are crucial to detect disease’s progression. This NDCP, supported by medical and paramedical teams from neuromuscular centres, provides a comprehensive overview of calpainopathy and its management approaches.

## Data Availability

The data analysed in this review are all from publicly available sources. Specific citations for the included studies can be found in the references section of this article.

## Abbreviations

AFM-Téléthon: French Muscular Dystrophy Association (Association Française Contre les Myopathies)
CAPN3: Calpain-3 (gene coding for the muscle-specific calpain-3 cysteine protease )
CK: Creatine Kinase
CT-Scan: Computerized Tomography or CT scan
ENMG: Electroneuromyography
MRI: Magnetic Resonance Imaging
LGMD R1 ex-LGMD 2A: Limb Girdle Muscular Dystrophy Recessive type 1 previously Limb Girdle Muscle Dystrophy type 2A
LGMD D4: Limb Girdle Muscular Dystrophy Dominant type 4
NGS: Next-Generation Sequencing
NDCP: National Diagnosis and Care Protocol
STIR (sequence): Short TI Inversion Recovery
WGS: Whole Genome Sequencing
PM&R: Physical Medicine and Rehabilitation
MFM: Motor Function Measure
NSAD: North Star Ambulatory Assessment
VUS: Variant of Uncertain Significance

## References

[1] Richard I, Broux O, Allamand V, Fougerousse F, Chiannilkulchai N, Bourg N, et al. Mutations in the proteolytic enzyme calpain 3 cause limb-girdle muscular dystrophy type 2A. Cell. 1995;81(1):27–40. 7720071 10.1016/0092-8674(95)90368-2

[2] Sáenz A, Leturcq F, Cobo AM, Poza JJ, Ferrer X, Otaegui D, et al. LGMD2A: genotype-phenotype correlations based on a large mutational survey on the calpain 3 gene. Brain. 2005;128(Pt 4):732–42. 15689361 10.1093/brain/awh408

[3] Angelini C. Calpainopathy. In: Adam MP, Feldman J, Mirzaa GM, Pagon RA, Wallace SE, Amemiya A, editors. GeneReviews^®^ [Internet]. Seattle (WA): University of Washington, Seattle; 1993 [cited 2025 Nov 12]. Available from: http://www.ncbi.nlm.nih.gov/books/NBK1313/20301490

[4] Schessl J, Walter MC, Schreiber G, Schara U, Müller CR, Lochmüller H, et al. Phenotypic variability in siblings with calpainopathy (LGMD2A). Acta Myol. 2008;27(2):54–8. 19364062 PMC2858935

[5] de Paula F, Vainzof M, Passos-Bueno MR, de Cássia M, Pavanello R, Matioli SR, Anderson VB. Clinical variability in calpainopathy: what makes the difference? Eur J Hum Genet. 2002;10(12):825–32. 12461690 10.1038/sj.ejhg.5200888

[6] Martinez-Thompson JM, Moore SA, Liewluck T. A novel CAPN3 mutation in late-onset limb-girdle muscular dystrophy with early respiratory insufficiency. J Clin Neurosci. 2018;53:229–31. 29685414 10.1016/j.jocn.2018.04.025 PMC6792001

[7] Hunn SM, Alfano LN, Jones A, Butler A, Lowes LP, Iammarino MA, et al. Clinical Trial Readiness in Limb Girdle Muscular Dystrophy R1 (LGMDR1): A GRASP Consortium Study. Ann Clin Transl Neurol. 2025;12(6):1179–86. 40237364 10.1002/acn3.70049 PMC12172116

[8] Richard I, Hogrel JY, Stockholm D, Payan CAM, Fougerousse F, Calpainopathy Study Group. Natural history of LGMD2A for delineating outcome measures in clinical trials. Ann Clin Transl Neurol. 2016;3(4):248–65. 27081656 10.1002/acn3.287 PMC4818744

[9] Fanin M, Angelini C. Protein and genetic diagnosis of limb girdle muscular dystrophy type 2A: The yield and the pitfalls. Muscle Nerve. 2015;52(2):163–73. 25900067 10.1002/mus.24682

[10] Estévez-Arias B, Segarra-Casas A, Romo L, Polavarapu K, O’Heir E, Singer-Berk M, et al. 490PA recurrent non-coding 3’UTR variant as a hidden second hit in 11 families with CAPN3-related limb girdle muscular dystrophy. Neuromuscul Disord. 2025;53:105786.

[11] Krag T, Nasho E, Brady L, Verebi C, Leturcq F, Malfatti E, et al. Variants in CAPN3 causing autosomal dominant limb-girdle muscular dystrophy combined with calpain-3 deficiency. Hum Mutat. 2025;2025:9301465. 40226307 10.1155/humu/9301465 PMC11972127

[12] Nallamilli BRR, Chakravorty S, Kesari A, Tanner A, Ankala A, Schneider T, et al. Genetic landscape and novel disease mechanisms from a large LGMD cohort of 4656 patients. Ann Clin Transl Neurol. 2018;5(12):1574–87. 30564623 10.1002/acn3.649 PMC6292381

[13] Martinez-Thompson JM, Niu Z, Tracy JA, Moore SA, Swenson A, Wieben ED, et al. Autosomal dominant calpainopathy due to heterozygous CAPN3 C.643_663del21. Muscle Nerve. 2018;57(4):679–83. 28881388 10.1002/mus.25970 PMC5915624

[14] Cerino M, Campana-Salort E, Salvi A, Cintas P, Renard D, Juntas Morales R, et al. Novel CAPN3 variant associated with an autosomal dominant calpainopathy. Neuropathol Appl Neurobiol. 2020;46(6):564–78. 32342993 10.1111/nan.12624

[15] Cerino M, Bartoli M, Riccardi F, Le Goanvic B, Blanck V, Salvi A, et al. Autosomal dominant segregation of CAPN3 c.598_612del15 associated with a mild form of calpainopathy. Ann Clin Transl Neurol. 2020;7(12):2538–40. 33107701 10.1002/acn3.51193 PMC7732236

[16] Mao B, Yang J, Zhao X, Jia X, Shi X, Zhao L, et al. Identification and functional characterization of a novel heterozygous splice–site mutation in the calpain 3 gene causes rare autosomal dominant limb–girdle muscular dystrophy. Exp Ther Med. 2024;27(3):97. 38356676 10.3892/etm.2024.12385 PMC10865457

[17] Massucco S, Fossa P, Fiorillo C, Faedo E, Gemelli C, Barresi R, et al. Case report: A single novel calpain 3 gene variant associated with mild myopathy. Front Genet. 2024;15:1437859. 39703226 10.3389/fgene.2024.1437859 PMC11655484

[18] González-Mera L, Ravenscroft G, Cabrera-Serrano M, Ermolova N, Domínguez-González C, Arteche-López A, et al. Heterozygous CAPN3 missense variants causing autosomal-dominant calpainopathy in seven unrelated families. Neuropathol Appl Neurobiol. 2021;47(2):283–96. 32896923 10.1111/nan.12663

[19] Malfatti E, Richard I. [Calpainopathies: state of the art and therapeutic perspectives]. Med Sci (Paris). 2020;36 Hors série n° 2:17–21. 10.1051/medsci/2020244 33427631

[20] Vissing J, Barresi R, Witting N, Van Ghelue M, Gammelgaard L, Bindoff LA, et al. A heterozygous 21-bp deletion in CAPN3 causes dominantly inherited limb girdle muscular dystrophy. Brain. 2016;139(Pt 8):2154–63. 27259757 10.1093/brain/aww133

[21] Richard I, Simental E, Staelens C, Eng C, Malfatti E. Le registre national des calpaïnopathies. Les Cahiers de Myologie. 2022;(25):40–1.

[22] Lostal W, Urtizberea JA, Richard I; Calpain 3 Study Group. 233rd ENMC International Workshop: Clinical trial readiness for calpainopathies, Naarden, The Netherlands, 15–17 September 2017. Neuromuscul Disord. 2018;28(6):540-549. 10.1016/j.nmd.2018.03.010 29655529

[23] Sorimachi H, Ishiura S, Suzuki K. Structure and physiological function of calpains. Biochem J. 1997;328(Pt 3):721–32. (Pt 3)(. 9396712 10.1042/bj3280721 PMC1218978

[24] Toral-Ojeda I, Aldanondo G, Lasa-Elgarresta J, Lasa-Fernández H, Fernández-Torrón R, de López A, et al. Calpain 3 deficiency affects SERCA expression and function in the skeletal muscle. Expert Rev Mol Med. 2016;18:e7. 27055500 10.1017/erm.2016.9 PMC4836212

[25] Lasa-Elgarresta J, Mosqueira-Martín L, Naldaiz-Gastesi N, Sáenz A, López de Munain A, Vallejo-Illarramendi A. Calcium Mechanisms in Limb-Girdle Muscular Dystrophy with CAPN3 Mutations. Int J Mol Sci. 2019;20(18):4548. 31540302 10.3390/ijms20184548 PMC6770289

[26] Duguez S, Bartoli M, Richard I. Calpain 3: a key regulator of the sarcomere? FEBS J. 2006;273(15):3427–36. 16884488 10.1111/j.1742-4658.2006.05351.x

[27] Beckmann JS, Spencer M. Calpain 3, the ‘gatekeeper’ of proper sarcomere assembly, turnover and maintenance. Neuromuscul Disord. 2008;18(12):913–21. 18974005 10.1016/j.nmd.2008.08.005 PMC2614824

[28] Kramerova I, Kudryashova E, Wu B, Germain S, Vandenborne K, Romain N, et al. Mitochondrial abnormalities, energy deficit and oxidative stress are features of calpain 3 deficiency in skeletal muscle. Hum Mol Genet. 2009;18(17):3194–205. 19483197 10.1093/hmg/ddp257 PMC2722983

[29] Valls A, Ruiz-Roldán C, Immanuel J, Alonso-Martín S, Gallardo E, Fernández-Torrón R, et al. The Role of Integrin β1D Mislocalization in the Pathophysiology of Calpain 3-Related Limb-Girdle Muscular Dystrophy. Cells. 2025;14(6):446. 40136695 10.3390/cells14060446 PMC11941428

[30] Protocoles Nationaux de Diagnostic et de Soins (PNDS). [Internet]. Haute Autorité de Santé. [cited 2025 Nov 24]. Available from: https://www.has-sante.fr/jcms/c_1340879/fr/protocoles-nationaux-de-diagnostic-et-de-soins-pnds

[31] Bardakov SN, Sorochanu I, Mkrtchyan LA, Slesarenko YS, Tsargush VA, Limaev IS, et al. Calpainopathy (limb-girdle muscular dystrophy type R1): clinical features, diagnostic approaches, and biotechnological treatment methods. J Neuromuscul Dis. 2025;12(5):594–618. 40452441 10.1177/22143602251345967 PMC13142882

[32] Spinazzi M, Poupiot J, Cassereau J, Leturcq F, Brunereau L, Malfatti E, et al. Late-onset camptocormia caused by a heterozygous in-frame CAPN3 deletion. Neuromuscul Disord. 2021;31(5):450–5. 33741228 10.1016/j.nmd.2021.02.012

[33] Fanin M, Nascimbeni AC, Aurino S, Tasca E, Pegoraro E, Nigro V, et al. Frequency of LGMD gene mutations in Italian patients with distinct clinical phenotypes. Neurology. 2009;72(16):1432–5. 19380703 10.1212/WNL.0b013e3181a1885e

[34] Hanisch F, Müller CR, Grimm D, Xue L, Traufeller K, Merkenschlager A, et al. Frequency of calpain-3 c.550delA mutation in limb girdle muscular dystrophy type 2 and isolated hyperCKemia in German patients. Clin Neuropathol. 2007;26(4):157–63. 17702496 10.5414/npp26157

[35] Barp A, Laforet P, Bello L, Tasca G, Vissing J, Monforte M, et al. European muscle MRI study in limb girdle muscular dystrophy type R1/2A (LGMDR1/LGMD2A). J Neurol. 2020;267(1):45–56. 31555977 10.1007/s00415-019-09539-y

[36] Aivazoglou LU, Guimarães JB, Costa MAF, Aihara AY, Cardoso FN, Pinto WBVDR, et al. Whole-Body MRI in Limb Girdle Muscular Dystrophy Type R1/2A: Correlation With Clinical Scores. Muscle Nerve. 2022;66(4):471–8. 35894554 10.1002/mus.27686

[37] Ilvnsjf C, V V, F C. Calpainopathy: Description of a novel mutation and clinical presentation with early severe contractures. Genes (Basel). 2020;11(2):E131. https://pubmed.ncbi.nlm.nih.gov/31991774/10.3390/genes11020129 PMC7074289 31991774

[38] Lahoria R, Milone M. Rhabdomyolysis featuring muscular dystrophies. J Neurol Sci. 2016;361:29–33. 26810512 10.1016/j.jns.2015.12.013

[39] de Albuquerque MAV, Neto A, Silva O, Zanoteli FMA, Reed E. UC. Limb-girdle muscular dystrophy type 2A in Brazilian children. Arq Neuropsiquiatr. 2015;73(12):993–7. 10.1590/0004-282X20150168 26677118

[40] Pollitt C, Anderson LV, Pogue R, Davison K, Pyle A, Bushby KM. The phenotype of calpainopathy: diagnosis based on a multidisciplinary approach. Neuromuscul Disord. 2001;11(3):287–96. 11297944 10.1016/s0960-8966(00)00197-8

[41] Burke G, Hillier C, Cole J, Sampson M, Bridges L, Bushby K, et al. Calpainopathy presenting as foot drop in a 41 year old. Neuromuscul Disord. 2010;20(6):407–10. 20580976 10.1016/j.nmd.2010.04.006

[42] Bérard C, Payan C, Hodgkinson I, Fermanian J, MFM Collaborative Study Group. A motor function measure for neuromuscular diseases. Construction and validation study. Neuromuscul Disord. 2005;15(7):463–70. 16106528 10.1016/j.nmd.2005.03.004

[43] James MK, Alfano LN, Muni-Lofra R, Reash NF, Sodhi J, Iammarino MA, et al. Validation of the North Star Assessment for Limb-Girdle Type Muscular Dystrophies. Phys Ther. 2022;102(10):pzac113. 35932452 10.1093/ptj/pzac113 PMC9586158

[44] Lue Y, Lin R, Chen S, Lu Y. Measurement of the Functional Status of Patients with Different Types of Muscular Dystrophy. Kaohsiung J Med Sci. 2009;25(6):325–33. 19560997 10.1016/S1607-551X(09)70523-6 PMC11917556

[45] Walton JN, Gardner-Medwin D, Edinburgh. 481–524. - References - Scientific Research Publishing [Internet]. [cited 2025 Nov 24]. Available from: https://www.scirp.org/reference/referencespapers?referenceid=2102187

[46] Pathak P, Sharma MC, Jha P, Sarkar C, Faruq M, Jha P, et al. Mutational Spectrum of CAPN3 with Genotype-Phenotype Correlations in Limb Girdle Muscular Dystrophy Type 2A/R1 (LGMD2A/LGMDR1) Patients in India. J Neuromuscul Dis. 2021;8(1):125–36. 33337384 10.3233/JND-200547

[47] Urtasun M, Sáenz A, Roudaut C, Poza JJ, Urtizberea JA, Cobo AM, et al. Limb-girdle muscular dystrophy in Guipúzcoa (Basque Country, Spain). Brain. 1998;121(Pt 9):1735–47. 9762961 10.1093/brain/121.9.1735

[48] Urinary N-. terminal titin fragment ascertained as biomarker in a small cohort of limb-girdle muscular dystrophy LGMDR1-calpain 3 related [Internet]. [cited 2026 Feb 6]. Available from: https://journals.sagepub.com/doi/epub/10.1177/22143602251323629 10.1177/22143602251323629 PMC13142826 40356353

[49] Mercuri E, Bushby K, Ricci E, Birchall D, Pane M, Kinali M, et al. Muscle MRI findings in patients with limb girdle muscular dystrophy with calpain 3 deficiency (LGMD2A) and early contractures. Neuromuscul Disord. 2005;15(2):164–71. 15694138 10.1016/j.nmd.2004.10.008

[50] Degardin A, Morillon D, Lacour A, Cotten A, Vermersch P, Stojkovic T. Morphologic imaging in muscular dystrophies and inflammatory myopathies. Skeletal Radiol. 2010;39(12):1219–27. 20449587 10.1007/s00256-010-0930-4

[51] Malfatti E. The Role of the Muscle Biopsy in the Era of Genetic Diagnosis. In: Narayanaswami P, Liewluck T, editors. Principles and Practice of the Muscular Dystrophies [Internet]. Cham: Springer International Publishing; 2023 [cited 2025 Nov 12]. pp. 255–67. Available from: 10.1007/978-3-031-44009-0_16

[52] Brown RH, Amato A. Calpainopathy and eosinophilic myositis. Ann Neurol. 2006;59(6):875–7. 16718709 10.1002/ana.20900

[53] Krahn M, Lopez de Munain A, Streichenberger N, Bernard R, Pécheux C, Testard H, et al. CAPN3 mutations in patients with idiopathic eosinophilic myositis. Ann Neurol. 2006;59(6):905–11. 16607617 10.1002/ana.20833

[54] Krahn M, Goicoechea M, Hanisch F, Groen E, Bartoli M, Pécheux C, et al. Eosinophilic infiltration related to CAPN3 mutations: a pathophysiological component of primary calpainopathy? Clin Genet. 2011;80(4):398–402. 21204801 10.1111/j.1399-0004.2010.01620.x

[55] Anderson LV, Harrison RM, Pogue R, Vafiadaki E, Pollitt C, Davison K, et al. Secondary reduction in calpain 3 expression in patients with limb girdle muscular dystrophy type 2B and Miyoshi myopathy (primary dysferlinopathies). Neuromuscul Disord. 2000;10(8):553–9. 11053681 10.1016/s0960-8966(00)00143-7

[56] Haravuori H, Vihola A, Straub V, Auranen M, Richard I, Marchand S, et al. Secondary calpain3 deficiency in 2q-linked muscular dystrophy: titin is the candidate gene. Neurology. 2001;56(7):869–77. 11294923 10.1212/wnl.56.7.869

[57] Milic A, Daniele N, Lochmüller H, Mora M, Comi GP, Moggio M, et al. A third of LGMD2A biopsies have normal calpain 3 proteolytic activity as determined by an in vitro assay. Neuromuscul Disord. 2007;17(2):148–56. 17236769 10.1016/j.nmd.2006.11.001

[58] Fanin M, Nascimbeni AC, Fulizio L, Trevisan CP, Meznaric-Petrusa M, Angelini C. Loss of calpain-3 autocatalytic activity in LGMD2A patients with normal protein expression. Am J Pathol. 2003;163(5):1929–36. 14578192 10.1016/S0002-9440(10)63551-1 PMC1892408

[59] Bouchard C, Tremblay JP. Portrait of Dysferlinopathy: Diagnosis and Development of Therapy. J Clin Med. 2023;12(18):6011. 37762951 10.3390/jcm12186011 PMC10531777

[60] Diaz-Manera J, Fernandez-Torron R, LLauger J, James MK, Mayhew A, Smith FE, et al. Muscle MRI in patients with dysferlinopathy: pattern recognition and implications for clinical trials. J Neurol Neurosurg Psychiatry. 2018;89(10):1071–81. 29735511 10.1136/jnnp-2017-317488 PMC6166612

[61] Villar Quiles RN, Richard I, Bouchet-Seraphin C, Stojkovic T. [Limb-Girdle Muscular Dystrophy type R9 linked to the FKRP gene: state of the art and therapeutic perspectives]. Med Sci (Paris). 2020;36:2:28–33. Hors série n°. 33427633 10.1051/medsci/2020239

[62] Xie Z, Xiao J, Zheng Y, Wang Z, Yuan Y. Magnetic Resonance Imaging Findings in the Muscle Tissue of Patients with Limb Girdle Muscular Dystrophy Type 2I Harboring the Founder Mutation c.545A > G in the FKRP Gene. Biomed Res Int. 2018;2018:3710814. 30003095 10.1155/2018/3710814 PMC5996470

[63] Vázquez J, Lefeuvre C, Escobar RE, Luna Angulo AB, Miranda Duarte A, Delia Hernandez A, et al. Phenotypic Spectrum of Myopathies with Recessive Anoctamin-5 Mutations. J Neuromuscul Dis. 2020;7(4):443–51. 32925086 10.3233/JND-200515

[64] de Bruyn A, Montagnese F, Holm-Yildiz S, Scharff Poulsen N, Stojkovic T, Behin A, et al. Anoctamin-5 related muscle disease: clinical and genetic findings in a large European cohort. Brain. 2023;146(9):3800–15. 36913258 10.1093/brain/awad088

[65] Vainzof M, Souza LS, Gurgel-Giannetti J, Zatz M. Sarcoglycanopathies: an update. Neuromuscul Disord. 2021;31(10):1021–7. 34404573 10.1016/j.nmd.2021.07.014

[66] Tasca G, Monforte M, Díaz-Manera J, Brisca G, Semplicini C, D’Amico A, et al. MRI in sarcoglycanopathies: a large international cohort study. J Neurol Neurosurg Psychiatry. 2018;89(1):72–7. 28889091 10.1136/jnnp-2017-316736

[67] Sacconi S, Camaño P, de Greef JC, Lemmers RJLF, Salviati L, Boileau P, et al. Patients with a phenotype consistent with facioscapulohumeral muscular dystrophy display genetic and epigenetic heterogeneity. J Med Genet. 2012;49(1):41–6. 21984748 10.1136/jmedgenet-2011-100101 PMC3560331

[68] Darras BT, Urion DK, Ghosh PS. Dystrophinopathies. In: Adam MP, Bick S, Mirzaa GM, Pagon RA, Wallace SE, Amemiya A, editors. GeneReviews^®^ [Internet]. Seattle (WA): University of Washington, Seattle; 1993 [cited 2025 Nov 27]. Available from: http://www.ncbi.nlm.nih.gov/books/NBK1119/20301298

[69] Arikawa E, Hoffman EP, Kaido M, Nonaka I, Sugita H, Arahata K. The frequency of patients with dystrophin abnormalities in a limb-girdle patient population. Neurology. 1991;41(9):1491–6. 1842672 10.1212/wnl.41.9.1491

[70] Shaboodien G, Watkins DA, Pillay K, Beighton P, Heckmann JM, Mayosi BM. Limb-girdle weakness in a marfanoid man: distinguishing calpainopathy from Becker’s muscular dystrophy. Pract Neurol. 2015;15(2):152–4. 25573340 10.1136/practneurol-2014-000992

[71] Pénisson-Besnier I, Richard I, Dubas F, Beckmann JS, Fardeau M. Pseudometabolic expression and phenotypic variability of calpain deficiency in two siblings. Muscle Nerve. 1998;21(8):1078–80. 9655129 10.1002/(sici)1097-4598(199808)21:8<1078::aid-mus15>3.0.co;2-q

[72] Krahn M, Biancalana V, Cerino M, Perrin A, Michel-Calemard L, Nectoux J, et al. A National French consensus on gene lists for the diagnosis of myopathies using next-generation sequencing. Eur J Hum Genet. 2019;27(3):349–52. 30552423 10.1038/s41431-018-0305-1 PMC6460575

[73] Ghaoui R, Cooper ST, Lek M, Jones K, Corbett A, Reddel SW, et al. Use of Whole-Exome Sequencing for Diagnosis of Limb-Girdle Muscular Dystrophy: Outcomes and Lessons Learned. JAMA Neurol. 2015;72(12):1424–32. 26436962 10.1001/jamaneurol.2015.2274

[74] Fardeau M, Hillaire D, Mignard C, Feingold N, Feingold J, Mignard D, et al. Juvenile limb-girdle muscular dystrophy. Clinical, histopathological and genetic data from a small community living in the Reunion Island. Brain. 1996;119(Pt 1):295–308. 8624690 10.1093/brain/119.1.295

[75] Richard I, Brenguier L, Dinçer P, Roudaut C, Bady B, Burgunder JM, et al. Multiple independent molecular etiology for limb-girdle muscular dystrophy type 2A patients from various geographical origins. Am J Hum Genet. 1997;60(5):1128–38. 9150160 PMC1712426

[76] Fardeau M, Eymard B, Mignard C, Tomé FM, Richard I, Beckmann JS. Chromosome 15-linked limb-girdle muscular dystrophy: clinical phenotypes in Reunion Island and French metropolitan communities. Neuromuscul Disord. 1996;6(6):447–53. 9027854 10.1016/s0960-8966(96)00387-2

[77] Norwood F, de Visser M, Eymard B, Lochmüller H, Bushby K, EFNS Guideline Task Force. EFNS guideline on diagnosis and management of limb girdle muscular dystrophies. Eur J Neurol. 2007;14(12):1305–12. 18028188 10.1111/j.1468-1331.2007.01979.x

[78] Angelini C, Nardetto L, Borsato C, Padoan R, Fanin M, Nascimbeni AC, et al. The clinical course of calpainopathy (LGMD2A) and dysferlinopathy (LGMD2B). Neurol Res. 2010;32(1):41–6. 20092694 10.1179/174313209X380847

[79] Zatz M, de Paula F, Starling A, Vainzof M. The 10 autosomal recessive limb-girdle muscular dystrophies. Neuromuscul Disord. 2003;13(7–8):532–44. 12921790 10.1016/s0960-8966(03)00100-7

[80] Gallardo E, Saenz A, Illa I. Limb-girdle muscular dystrophy 2A. Handb Clin Neurol. 2011;101:97–110. 21496626 10.1016/B978-0-08-045031-5.00006-2

[81] Fanin M, Nascimbeni AC, Angelini C. Gender difference in limb-girdle muscular dystrophy: a muscle fiber morphometric study in 101 patients. Clin Neuropathol. 2014;33(3):179–85. 24618072 10.5414/NP300728

[82] Piluso G, Politano L, Aurino S, Fanin M, Ricci E, Ventriglia VM, et al. Extensive scanning of the calpain-3 gene broadens the spectrum of LGMD2A phenotypes. J Med Genet. 2005;42(9):686–93. 16141003 10.1136/jmg.2004.028738 PMC1736133

[83] Bennell KL, Marshall CJ, Dobson F, Kasza J, Lonsdale C, Hinman RS. Does a Web-Based Exercise Programming System Improve Home Exercise Adherence for People With Musculoskeletal Conditions? A Randomized Controlled Trial. Am J Phys Med Rehabil. 2019;98(10):850–8. 31021823 10.1097/PHM.0000000000001204

[84] Siciliano G, Simoncini C, Giannotti S, Zampa V, Angelini C, Ricci G. Muscle exercise in limb girdle muscular dystrophies: pitfall and advantages. Acta Myol. 2015;34(1):3–8. 26155063 PMC4478773

[85] Mori-Yoshimura M, Segawa K, Minami N, Oya Y, Komaki H, Nonaka I, et al. Cardiopulmonary dysfunction in patients with limb-girdle muscular dystrophy 2A. Muscle Nerve. 2017;55(4):465–9. 27500519 10.1002/mus.25369 PMC5396288

[86] Dirik E, Aydin A, Kurul S, Sahin B. Limb girdle muscular dystrophy type 2A presenting with cardiac arrest. Pediatr Neurol. 2001;24(3):235–7. 11301229 10.1016/s0887-8994(00)00262-9

[87] De Vito EL. [When should and when should not use oxygen in neuromuscular diseases?]. Med (B Aires). 2022;82(2):244–8. 35417389

[88] Tobin MJ, Jubran A. Oxygen takes the breath away: old sting, new setting. Mayo Clin Proc. 1995;70(4):403–4. 7898152 10.4065/70.4.403

[89] Mendell JR, Muntoni F, McDonald CM, Mercuri EM, Ciafaloni E, Komaki H, et al. AAV gene therapy for Duchenne muscular dystrophy: the EMBARK phase 3 randomized trial. Nat Med. 2025;31(1):332–41. 39385046 10.1038/s41591-024-03304-z PMC11750718

[90] Bartoli M, Roudaut C, Martin S, Fougerousse F, Suel L, Poupiot J, et al. Safety and efficacy of AAV-mediated calpain 3 gene transfer in a mouse model of limb-girdle muscular dystrophy type 2A. Mol Ther. 2006;13(2):250–9. 16290124 10.1016/j.ymthe.2005.09.017

[91] Roudaut C, Le Roy F, Suel L, Poupiot J, Charton K, Bartoli M, et al. Restriction of calpain3 expression to the skeletal muscle prevents cardiac toxicity and corrects pathology in a murine model of limb-girdle muscular dystrophy. Circulation. 2013;128(10):1094–104. 23908349 10.1161/CIRCULATIONAHA.113.001340

[92] Lostal W, Roudaut C, Faivre M, Charton K, Suel L, Bourg N, et al. Titin splicing regulates cardiotoxicity associated with calpain 3 gene therapy for limb-girdle muscular dystrophy type 2A. Sci Transl Med. 2019;11(520):eaat6072. 31776291 10.1126/scitranslmed.aat6072 PMC7397529

[93] Shoti J, Qing K, Keeler GD, Duan D, Byrne BJ, Srivastava A. Development of capsid- and genome-modified optimized AAVrh74 vectors for muscle gene therapy. Mol Ther Methods Clin Dev. 2023;31:101147. 38046199 10.1016/j.omtm.2023.101147 PMC10690633

[94] Müthel S, Marg A, Ignak B, Kieshauer J, Escobar H, Stadelmann C, et al. Cas9-induced single cut enables highly efficient and template-free repair of a muscular dystrophy causing founder mutation. Mol Ther Nucleic Acids. 2023;31:494–511. 36865086 10.1016/j.omtn.2023.02.005 PMC9972404

[95] Lasa-Elgarresta J, Mosqueira-Martín L, González-Imaz K, Marco-Moreno P, Gerenu G, Mamchaoui K, et al. Targeting the Ubiquitin-Proteasome System in Limb-Girdle Muscular Dystrophy With CAPN3 Mutations. Front Cell Dev Biol. 2022;10:822563. 35309930 10.3389/fcell.2022.822563 PMC8924035

[96] Jahnke VE, Peterson JM, Van Der Meulen JH, Boehler J, Uaesoontrachoon K, Johnston HK, et al. Mitochondrial dysfunction and consequences in calpain-3-deficient muscle. Skelet Muscle. 2020;10(1):37. 33308300 10.1186/s13395-020-00254-1 PMC7730798

[97] Rico A, Guembelzu G, Palomo V, Martínez A, Aiastui A, Casas-Fraile L, et al. Allosteric Modulation of GSK-3β as a New Therapeutic Approach in Limb Girdle Muscular Dystrophy R1 Calpain 3-Related. Int J Mol Sci. 2021;22(14):7367. 34298987 10.3390/ijms22147367 PMC8308041

[98] Bruge C, Bourg N, Pellier E, Tournois J, Polentes J, Benabides M, et al. High-throughput screening identifies bazedoxifene as a potential therapeutic for dysferlin-deficient limb girdle muscular dystrophy. Br J Pharmacol. 2025;182(13):2930–49. 40108832 10.1111/bph.70017

[99] Hoch L, Bourg N, Degrugillier F, Bruge C, Benabides M, Pellier E, et al. Dual Blockade of Misfolded Alpha-Sarcoglycan Degradation by Bortezomib and Givinostat Combination. Front Pharmacol. 2022;13:856804. 35571097 10.3389/fphar.2022.856804 PMC9093689

[100] Wagner KR, Fleckenstein JL, Amato AA, Barohn RJ, Bushby K, Escolar DM, et al. A phase I/IItrial of MYO-029 in adult subjects with muscular dystrophy. Ann Neurol. 2008;63(5):561–71. 18335515 10.1002/ana.21338

